# Predicting the Functional Effect of Amino Acid Substitutions and Indels

**DOI:** 10.1371/journal.pone.0046688

**Published:** 2012-10-08

**Authors:** Yongwook Choi, Gregory E. Sims, Sean Murphy, Jason R. Miller, Agnes P. Chan

**Affiliations:** The J. Craig Venter Institute, Rockville, Maryland, United States of America; UMR-S665, INSERM, Université Paris Diderot, INTS, France

## Abstract

As next-generation sequencing projects generate massive genome-wide sequence variation data, bioinformatics tools are being developed to provide computational predictions on the functional effects of sequence variations and narrow down the search of casual variants for disease phenotypes. Different classes of sequence variations at the nucleotide level are involved in human diseases, including substitutions, insertions, deletions, frameshifts, and non-sense mutations. Frameshifts and non-sense mutations are likely to cause a negative effect on protein function. Existing prediction tools primarily focus on studying the deleterious effects of single amino acid substitutions through examining amino acid conservation at the position of interest among related sequences, an approach that is not directly applicable to insertions or deletions. Here, we introduce a versatile alignment-based score as a new metric to predict the damaging effects of variations not limited to single amino acid substitutions but also in-frame insertions, deletions, and multiple amino acid substitutions. This alignment-based score measures the change in sequence similarity of a query sequence to a protein sequence homolog before and after the introduction of an amino acid variation to the query sequence. Our results showed that the scoring scheme performs well in separating disease-associated variants (n = 21,662) from common polymorphisms (n = 37,022) for UniProt human protein variations, and also in separating deleterious variants (n = 15,179) from neutral variants (n = 17,891) for UniProt non-human protein variations. In our approach, the area under the receiver operating characteristic curve (AUC) for the human and non-human protein variation datasets is ∼0.85. We also observed that the alignment-based score correlates with the deleteriousness of a sequence variation. In summary, we have developed a new algorithm, PROVEAN (**Pro**tein **V**ariation **E**ffect **An**alyzer), which provides a generalized approach to predict the functional effects of protein sequence variations including single or multiple amino acid substitutions, and in-frame insertions and deletions. The PROVEAN tool is available online at http://provean.jcvi.org.

## Introduction

Recent advances in high-throughput technologies have generated massive amounts of genome sequence and genotype data for humans and a number of model species. Approximately 15 million single nucleotide variations and one million short indels (insertions and deletions) of the human population have been cataloged as a result of the International HapMap Project and the ongoing 1000 Genomes Project [1], [2]. Additional large-scale projects targeting human cancers and common human diseases have further expanded the list of mutations found in healthy and diseased individuals [3]. Results from the 1000 Genomes project suggest that each individual human genome typically carries approximately 10,000–11,000 non-synonymous and 10,000–12,000 synonymous variations [1], [4]. In addition, an individual is estimated to carry 200 small in-frame indels and is heterozygous for 50–100 disease-associated variants as defined by the Human Gene Mutation Database [1].

The enormous amount of sequence variation data generated from large-scale projects necessitates computational approaches to assess the potential impact of amino acid changes on gene functions. Most computational prediction tools for amino acid variants rely on the assumption that protein sequences observed among living organisms have survived natural selection. Therefore evolutionarily conserved amino acid positions across multiple species are likely to be functionally important, and amino acid substitutions observed at conserved positions will potentially lead to deleterious effects on gene functions. A number of computational methods have been developed based on such evolutionary principles to predict the effect of coding variants on protein function, including SIFT [5], PolyPhen-2 [6], Mutation Assessor [7], MAPP [8], PANTHER [9], LogR.E-value [10], Condel [11] and several others [12], [13]. In general, the prediction tools obtain information on amino acid conservation directly from alignment with homologous and distantly related sequences. SIFT computes a combined score derived from the distribution of amino acid residues observed at a given position in the sequence alignment and the estimated unobserved frequencies of amino acid distribution calculated from a Dirichlet mixture. PolyPhen-2 uses a naïve Bayes classifier to utilize information derived from sequence alignments and protein structural properties (e.g. accessible surface area of amino acid residue, crystallographic beta-factor, etc.). Mutation Assessor captures the evolutionary conservation of a residue in a protein family and its subfamilies using combinatorial entropy measurement. MAPP derives information from the physicochemical constraints of the amino acid of interest (e.g. hydropathy, polarity, charge, side-chain volume, free energy of alpha-helix or beta-sheet). PANTHER PSEC (position-specific evolutionary conservation) scores are computed based on PANTHER Hidden Markov Model families. LogR.E-value prediction is based on a change in the E-value caused by an amino acid substitution obtained from the sequence homology HMMER tool based on Pfam domain models. Finally, Condel provides a method to produce a combined prediction result by integrating the scores obtained from different predictive tools.

To the best of our knowledge most prediction tools focus on single amino acid substitutions and therefore are not able to deal with sequence variations such as amino acid insertions, deletions, and multiple amino acid substitutions [13]. In addition to single amino acid substitutions, there are other variation classes associated with disease phenotypes. For example, a common disease variant associated with the genetic disease cystic fibrosis is a deletion of phenylalanine at position 508, part of the ATP-binding domain of the CFTR protein. The prevalence of the ΔF508 allele in cystic fibrosis patients was 71% [14], [15]. In the Human Gene Mutation Database (Professional ver2011.3), at the gene sequence level approximately half of the human disease variations are associated with single nucleotide substitutions (57%), and close to one-fourth of disease mutations (22%) are associated with small indels [16], [17].

Here we present a new algorithm, PROVEAN (Protein Variation Effect Analyzer), which predicts the functional impact for all classes of protein sequence variations not only single amino acid substitutions but also insertions, deletions, and multiple substitutions. We tested our method on a large set of human and non-human protein variations obtained from the UniProtKB/Swiss-Prot database and experimental datasets previously generated from mutagenesis experiments for the human tumor suppressor protein TP53 and the ATP-binding cassette transporter 1 protein ABCA1 [18], [19]. Our results show that the predictive ability of PROVEAN for single amino acid substitution is highly comparable to other popular leading tools. Most importantly, the PROVEAN algorithm is also capable of handling in-frame insertion, deletions, and multiple substitutions with equally high performance and accuracy of prediction. In addition, we also show that the PROVEAN scores correlate with biological activity level and may be used as an indicator for the degree of functional impact of a protein variation.

## Results

### Delta alignment score

In pairwise sequence alignments, alignment scores can be used as a measure of sequence similarity to assess how likely the sequence pairs are homologous or related. In keeping with this idea, one can interpret a change in the alignment score caused by an amino acid variation as the impact of the variation on protein function. Specifically, given a protein A, let us assume there is a homologous protein B which is functional. To measure the effect of a variation on protein A, we can measure the similarity of protein A to B before and after the introduction of the variation. Our assumption is that a variation that reduces the similarity of protein A to the functional homolog protein B is more likely to cause a damaging effect. For this purpose, we suggest a change in the “alignment score” to be used as a measure of change in “similarity” caused by a variation.

To quantify the degree of impact of a variation on protein function, we define a *delta alignment score* (or simply *delta score*) of a protein query sequence 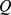 and its variation 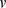 with respect to another protein subject sequence 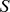 as the change in semi-global alignment score (i.e., no penalty on end gaps in global alignment [20]) between 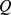 and 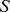 caused by 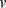. More formally,
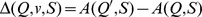
where 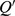 is the variant sequence of 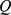 caused by 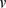, and 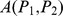 is the semi-global alignment score between two protein sequences 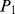 and 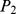, which is computed based on a given amino acid substitution matrix (e.g. BLOSUM62) and gap penalties.

**Figure 1:**
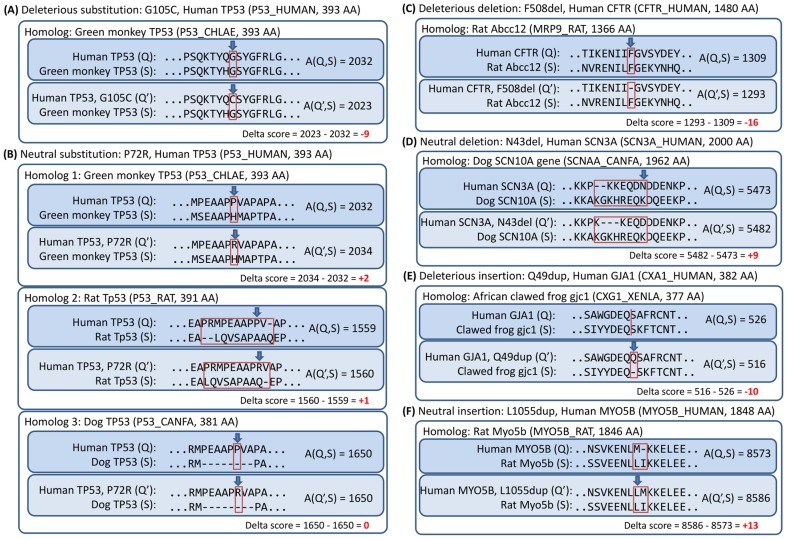
Examples of computing and interpreting delta alignment scores for six different known variations, (A) deleterious substitution (MIM:151623), (B) neutral substitution (dbSNP:rs1042522), (C) deleterious deletion (MIM:219700), (D) neutral deletion (dbSNP:rs72471101), (E) deleterious insertion (MIM:164200), and (F) neutral insertion (dbSNP:rs10625857) with respect to the selected homologous proteins. The amino acid residue replaced, deleted, or inserted is indicated by an arrow, and the difference between two alignments is indicated by a rectangle. Low delta scores are interpreted as deleterious, and high delta scores are interpreted as neutral. The BLOSUM62 and gap penalties of 10 for opening and 1 for extension were used.

The delta score can be used to measure the effect of a variation. That is, low delta scores are interpreted as amino acid variations leading to a deleterious effect on protein function (Figure 1A, C, and E), while high delta scores are interpreted as variations with neutral effect on protein function (Figure 1B, D, and F). Since the delta score is computed from alignment scores and that the alignment scores are computed based on a substitution matrix, the delta score approach has advantages over other tools as described below.

First, the delta score approach naturally utilizes a substitution matrix which implicitly captures information on the substitution frequency and chemical properties of 20 amino acid residues. Given a single amino acid substitution, if the reference amino acid residue is found to be conserved or similar to the aligned amino acid in a homologous sequence and that the frequency of substitution from the reference residue to the variant residue in question is low based on the substitution matrix score, then the substitution will produce a low delta score which suggests a deleterious effect of the substitution (Figure 1A). Conversely, if the variant amino acid residue instead of the reference residue is found to be similar to the aligned amino acid in the homologous sequence, then the substitution will produce a high delta score to suggest a neutral effect of the variation (Figure 1B, Homolog 1).

Second, the delta score is not only determined by the amino acid position where the variation is observed but can also be determined by the neighborhood that surrounds the site of variation (i.e., sequence context). The delta score is computed from alignment scores that encompass regions flanking both sides of the site of variation. In the scenario when an amino acid variation does not cause a change in the flanking sequence alignment (e.g. in ungapped regions, Figure 1A and B, Homolog 1), the delta score is simply determined by looking up two values from the substitution matrix scores and computing their differences (e.g. a BLOSUM62 score of “6” for a G→G change and a score of “-3” for a C→G change as shown in Figure 1A). In a different scenario when an amino acid variation causes a change in the sequence alignment in the neighborhood area of the site of variation (e.g. in gapped regions, Figure 1B, Homolog 2) or when the neighborhood area is aligned with gaps (Figure 1B, Homolog 3), the delta score is determined by the alignment scores derived from the flanking regions. In such cases, existing tools which base on frequency distribution or identity count of the aligned amino acids can be misled by the poorly aligned residues in a gapped alignment (Figure 1B, Homolog 2), or simply cannot make use of the homologous protein alignment because no amino acid can be aligned to derive count statistics (Figure 1B, Homolog 3).

**Figure 2:**
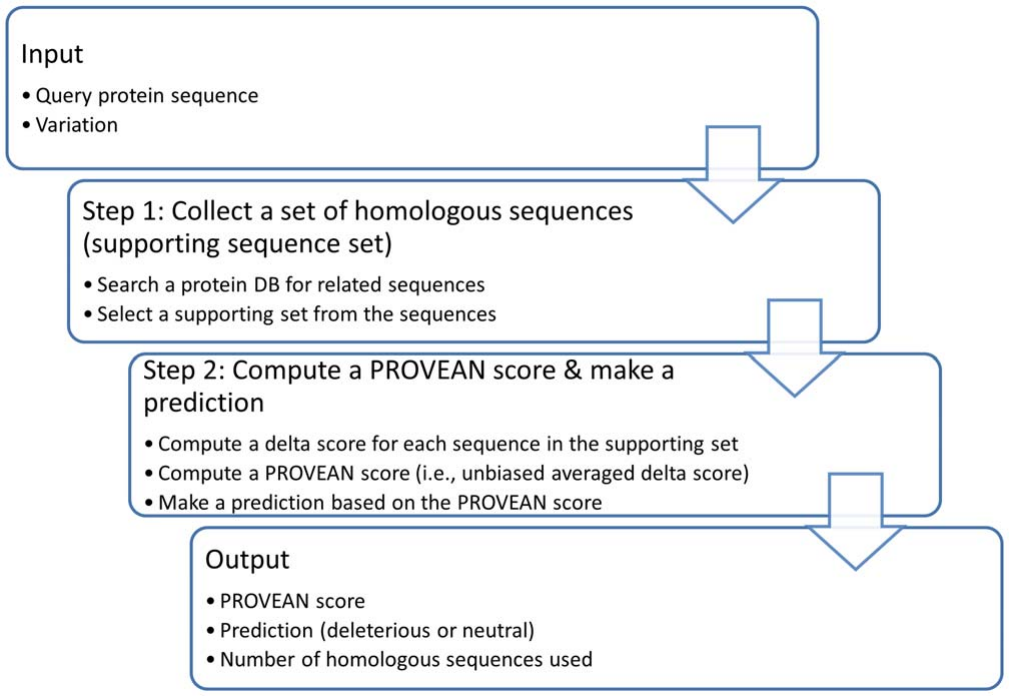
An overview of the PROVEAN procedure.

Finally, the most important advantage of our method is that the delta score approach considers alignment scores derived from the neighborhood regions and therefore can be directly extended to all classes of sequence variations including indels and multiple amino acid replacements. That is, the delta scores for other types of amino acid variations are computed in the same way as for single amino acid substitutions. In the case of amino acid insertion or deletion, the amino acids are inserted into or removed respectively from the variant sequence prior to performing the pair-wise sequence alignment and computing the alignment scores and delta score (Figure 1C–F). Using the delta alignment score approach, PROVEAN was developed to predict the effect of amino acid variations on protein function. An overview of the PROVEAN procedure is shown in Figure 2. The algorithm consists of (1) collection of homologous sequences, and (2) computation of an “unbiased averaged delta score” for making a prediction (See Methods for details). As an example, PROVEAN scores were computed for the human protein TP53 for all possible single amino acid substitutions, deletions, and insertions along the entire length of the protein sequence to demonstrate that PROVEAN scores indeed reflect and negatively correlate with amino acid conservation (Figure S1).

### New prediction tool PROVEAN

To test the predictive ability of PROVEAN, reference datasets were obtained from annotated protein variations available from the UniProtKB/Swiss-Prot database. For single amino acid substitutions, the “Human Polymorphisms and Disease Mutations” dataset (Release 2011_09) was used (will be referred to as the “humsavar”). In this dataset, single amino acid substitutions have been classified as disease variants (n = 20,821), common polymorphisms (n = 36,825), or unclassified. For the reference dataset, we assumed that the human disease variants will have deleterious effects on protein function and common polymorphisms will have neutral effects. Since the UniProt humsavar dataset only contains single amino acid substitutions, additional types of natural variation, including deletions, insertions, and replacements (in-frame substitution of multiple amino acids) of length up to 6 amino acids, were collected from the UniProtKB/Swiss-Prot database. Each variant in this dataset was annotated in-house as deleterious, neutral, or unknown based on keywords found in the description provided in the UniProt record (see Methods). A total of 729, 171, and 138 human protein variations of deletions, insertions, and replacements were collected, respectively. The number of UniProt human protein variants used in the predictability test is shown in Table 1.

**Table 1:** Number of human protein variations collected from the UniProt/Swiss-Prot database.

Variation type	Deleterious	Neutral	Total
Single amino acid substitutions	20821	36825	57646
Deletions	652	77	729
Insertions	110	61	171
Replacements	79	59	138
Total	21662	37022	58684

**Figure 3:**
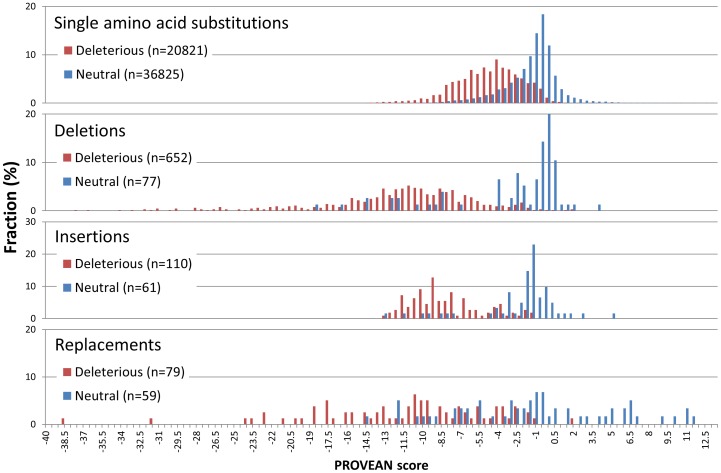
PROVEAN score distribution for deleterious and neutral human protein variations. For all classes of variations including substitutions, indels, and replacements, the distribution shows a distinct separation between the deleterious and neutral variations.

The PROVEAN tool was applied to the above dataset to generate a PROVEAN score for each variant. As shown in Figure 3, the score distribution shows a distinct separation between the deleterious and neutral variants for all classes of variations. This result shows that the PROVEAN score can be used as a measure to distinguish disease variants and common polymorphisms.

To optimize the predictive ability of PROVEAN for binary classification (the classification property is being deleterious), a PROVEAN score threshold was chosen to allow for the best balanced separation between the deleterious and neutral classes, that is, a threshold that maximizes the minimum of sensitivity and specificity. In the UniProt human variant dataset described above, the maximum balanced separation is achieved at the score threshold of −2.282. With this threshold the overall balanced accuracy was 79% (i.e., the average of sensitivity and specificity) (Table 2). The balanced separation and balanced accuracy were used so that threshold selection and performance measurement will not be affected by the sample size difference between the two classes of deleterious and neutral variations. The default score threshold and other parameters for PROVEAN (e.g. sequence identity for clustering, number of clusters) were determined using the UniProt human protein variant dataset (see Methods).

**Table 2:** Prediction accuracy for the UniProt human protein variations given a PROVEAN score threshold of −2.282.

Variation type	Sensitivity	Specificity	Balanced accuracy
Single amino acid substitutions	78.39	79.11	78.75
Deletions	95.86	67.53	81.70
Insertions	92.73	80.33	86.53
Replacements	92.41	61.02	76.71
Total	79.04	79.06	79.05

To determine whether the same parameters can be used generally, non-human protein variants available in the UniProtKB/Swiss-Prot database including viruses, fungi, bacteria, plants, etc. were collected. Each non-human variant was annotated in-house as deleterious, neutral, or unknown based on keywords in descriptions available in the UniProt record. When applied to our UniProt non-human variant dataset, the balanced accuracy of PROVEAN was about 77%, which is as high as that obtained with the UniProt human variant dataset (Table 3).

**Table 3:** Number of the UniProt non-human protein variations and prediction accuracy given a PROVEAN score threshold of −2.282.

	Number of variations			Accuracy		
Variation type	Deleterious	Neutral	Total	Sensitivity	Specificity	Balanced accuracy
Single amino acid substitutions	14117	16498	30615	80.22	75.33	77.75
Deletions	142	227	369	83.10	60.35	71.73
Insertions	34	137	171	76.47	73.72	75.10
Replacements	886	1029	1915	86.46	62.88	74.67
Total	15179	17891	33070	80.60	74.36	77.48

**Figure 4:**
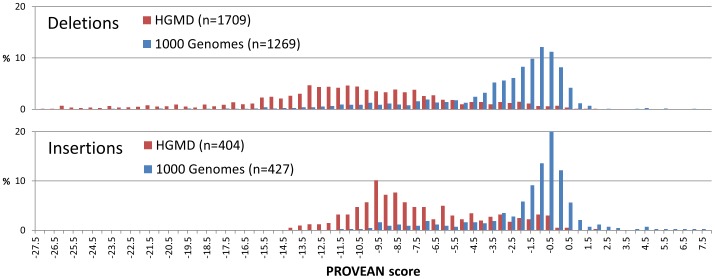
PROVEAN score distribution of deletions and insertions collected from the Human Gene Mutation Database (HGMD) and the 1000 Genomes Project. Only mutations annotated as “disease-causing” were collected from the HGMD. The distribution shows a distinct separation between the two datasets.

As an additional validation of the PROVEAN parameters and score threshold, indels of length up to 6 amino acids were collected from the Human Gene Mutation Database (HGMD) and the 1000 Genomes Project (Table 4, see Methods). The HGMD and 1000 Genomes indel dataset provides additional validation since it is more than four times larger than the human indels represented in the UniProt human protein variant dataset (Table 1), which were used for parameter selection. The average and median allele frequencies of the indels collected from the 1000 Genomes were 10% and 2%, respectively, which are high compared to the normal cutoff of 1–5% for defining common variations found in the human population. Therefore, we expected that the two datasets HGMD and 1000 Genomes will be well separated using the PROVEAN score with the assumption that the HGMD dataset represents disease-causing mutations and the 1000 Genomes dataset represents common polymorphisms. As expected, the indel variants collected from the HGMD and 1000 genome datasets showed a different PROVEAN score distribution (Figure 4). Using the default score threshold (−2.282), the majority of HGMD indel variants were predicted as deleterious, which included 94.0% of deletion variants and 87.4% of insertion variants. In contrast, for the 1000 Genome dataset, a much lower fraction of indel variants was predicted as deleterious, which included 40.1% of deletion variants and 22.5% of insertion variants.

**Table 4:** Number of deletions and insertions collected from the Human Gene Mutation Database and the 1000 Genomes Project and used for validation of PROVEAN.

	HGMD		1000 Genomes	
Length (in AA)	Deletion	Insertion	Deletion	Insertion
1	1103	174	1007	311
2	185	75	103	71
3	164	64	59	28
4	105	28	58	9
5	73	31	25	3
6	79	32	17	5
Total	1709	404	1269	427

### Comparison with other tools for single amino acid substitutions

Many tools exist to predict the damaging effects of single amino acid substitutions, but PROVEAN is the first to assess multiple types of variation including indels. Here we compared the predictive ability of PROVEAN for single amino acid substitutions with existing tools (SIFT, PolyPhen-2, and Mutation Assessor). For this comparison, we used the datasets of UniProt human and non-human protein variants, which were introduced in the previous section, and experimental datasets from mutagenesis experiments previously carried out for the *E.coli* LacI protein and the human tumor suppressor TP53 protein.

**Figure 5:**
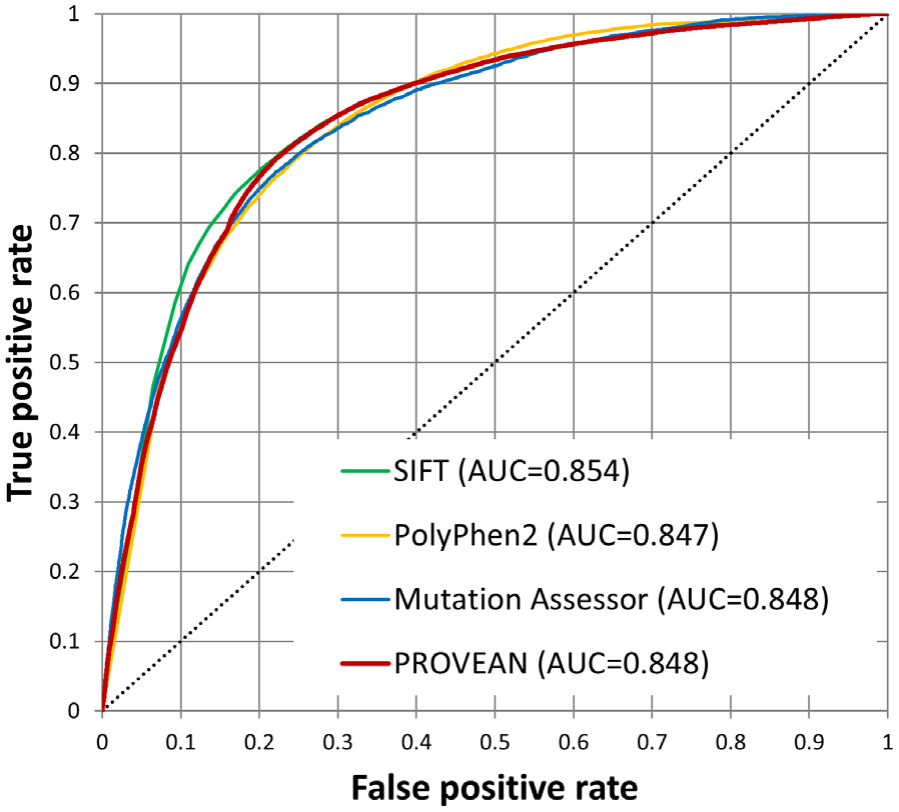
ROC curves of four different prediction tools for single amino acid substitutions found in human and non-human proteins. All tools show a similar predictive ability with the AUC value of ∼0.85.

For the combined UniProt human and non-human protein variant datasets containing 57,646 human and 30,615 non-human single amino acid substitutions, PROVEAN shows a performance similar to the three prediction tools tested. In the ROC (Receiver Operating Characteristic) analysis, the AUC (Area Under Curve) values for all tools including PROVEAN are ∼0.85 (Figure 5). The performance accuracy for the human and non-human datasets was computed based on the prediction results obtained from each tool (Table 5, see Methods). As shown in Table 5, for single amino acid substitutions, PROVEAN performs as well as other prediction tools tested. PROVEAN achieved a balanced accuracy of 78–79%. As noted in the column of “No prediction”, unlike other tools which may fail to provide a prediction in cases when only few homologous sequences exist or remain after filtering, PROVEAN can still provide a prediction because a delta score can be computed with respect to the query sequence itself even if there is no other homologous sequence in the supporting sequence set.

**Table 5:** Binary classification performance of four different tools for single amino acid substitutions in human and non-human proteins.

		Human dataset				Non-human dataset			
Tool	Threshold	Balanced accuracy	Sensitivity	Specificity	No prediction	Balanced accuracy	Sensitivity	Specificity	No prediction
PROVEAN	−2.282	78.75	78.39	79.11	0	77.75	80.22	75.27	0
Mutation Assessor	0.800	68.57	96.54	40.59	317 (0.55%)	69.15	93.17	45.13	732 (2.39%)
	1.900	78.15	85.29	71.02		74.23	81.30	67.16	
SIFT	0.050	76.99	85.03	68.95	1147 (1.99%)	78.36	87.45	69.27	1539 (5.03%)
PolyPhen-2	0.432	75.56	88.68	62.45	2279 (3.95%)	76.79	87.77	65.81	1499 (4.90%)

“Balanced accuracy” is a simple average of sensitivity and specificity, that is, (sensitivity+specificity)/2. The “No prediction” column shows the number of variants for which the tool fails to provide a prediction.

**Figure 6:**
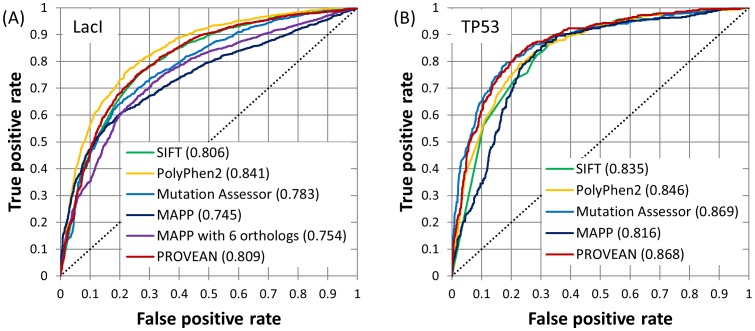
ROC curves of different prediction tools for single amino acid substitutions in (A) *E. coli* lac repressor protein and (B) human TP53 tumor suppressor protein. The AUC values are shown in the legend. The top two performers for LacI were PolyPhen-2 and PROVEAN, and those for TP53 were Mutation Assessor and PROVEAN.

We also compared PROVEAN with other prediction tools using two experimental datasets obtained from mutagenesis experiments that had been performed on LacI and TP53 (see Methods). Figure 6 shows that PROVEAN provides a good overall performance consistently and is among the top two best performers for both datasets based on the AUC values.

In addition, we also tested Condel, a tool which provides a consensus prediction by combining results from multiple prediction tools, for the human protein variant dataset used in this study (see Methods). Condel provided a balanced accuracy of 70% and 76% respectively, when using the published default threshold (0.469) and a threshold selected to maximize the balanced accuracy (0.790) (Table S1).

### Delta score correlates with biological activity of TP53 and ABCA1 variations

In addition to binary classification, we also investigated if the PROVEAN score can be used for predicting the degree of deleteriousness of a protein variation. Two experimental datasets obtained from the TP53 and ABCA1 (ATP-binding cassette transporter 1 protein) genes were examined, both of which also included the corresponding functional activity levels measured for each mutation found in the protein sequence [18], [19].

**Figure 7:**
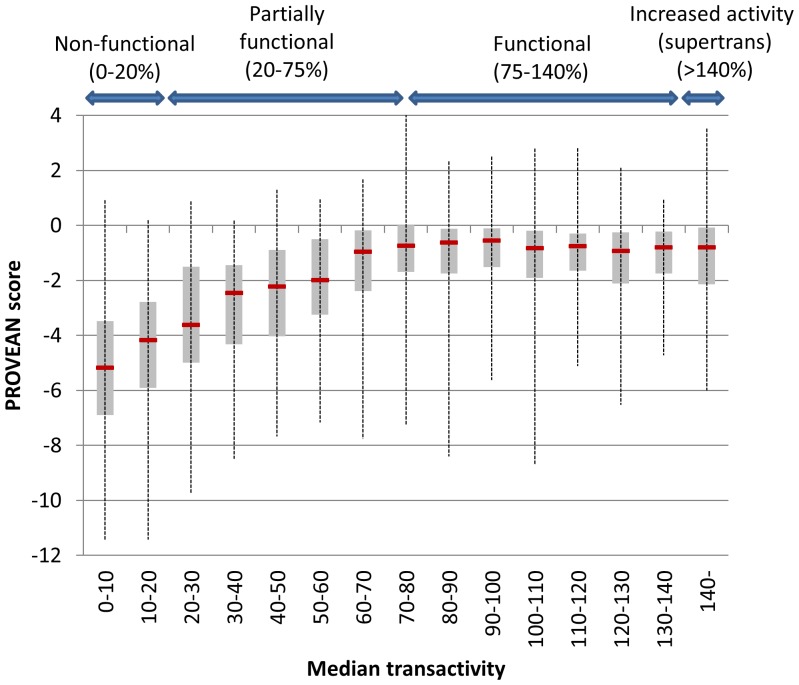
PROVEAN score distribution of TP53 variations binned into 15 classes based on transactivation levels. For each class, a box plot is shown. The vertical line shows the whole range of delta scores, the thick horizontal line shows the median, and the gray rectangle shows the interquartile range (25%–75%). The PROVEAN score increases and correlates with median transactivation level for the non-functional and partially functional classes of variations.

For the TP53 variation dataset, the single amino acid variations were divided into 15 classes based on a functional assay which measured the median transactivation level of the mutant protein. The distribution of the PROVEAN scores was computed for each class. As shown in Figure 7 and Figure S2, the PROVEAN score increases and correlates with the reported transactivation level, especially for those classified as either non-functional or partially functional.

**Figure 8:**
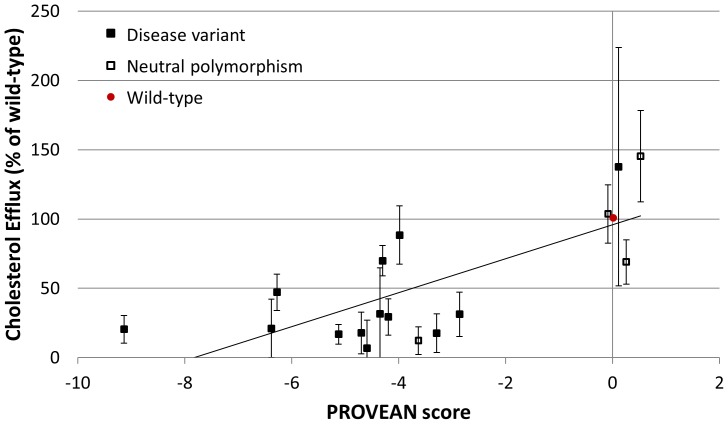
Correlation of cholesterol efflux values with the PROVEAN scores for ABCA1 variations. In general, an increase in score (i.e. less deleterious effects) correlates with an increase in cholesterol efflux activity.

For the ABCA1 variation dataset, cholesterol efflux was measured in 17 mutants and wild-type to assess ABCA1 functional activity. PROVEAN scores were generated for all ABCA1 mutants. A total of 15 mutants (88%) were correctly predicted by PROVEAN with reference to the UniProt disease versus common polymorphism classification of the amino acid variants (Table S2). Figure 8 shows that the PROVEAN score increases and correlates with the level of cholesterol efflux (Pearson's correlation coefficient of 0.74). In general, an increase in score correlates with an increased cholesterol efflux activity.

## Discussion

Most existing prediction tools for coding variants extract information from alignments with homologous sequences to define amino acid residues and positions that are evolutionarily conserved and therefore likely to be functionally important. In this case, amino acid variants which deviate from the frequently occurring residues are predicted as deleterious to protein function. In addition to information such as amino acid frequency distribution, log-odd scores from amino acid substitution matrices have also been used as a metric to measure amino acid conservation, and amino acid variants which correspond to non-conserved substitutions are predicted as deleterious [21], [22]. However, in all of the above approaches sequence alignment information from the neighborhood amino acid residues surrounding the position of interest is not directly taken into account to compute the impact of amino acid variations on protein function. Our approach introduced a region-based “delta alignment score” which measures the impact of an amino acid variation not only based on the amino acid residue at the position of interest but also the quality of sequence alignment derived from the neighborhood flanking sequences. Because of the unique property of the scoring scheme, the new approach can provide functional predictions to assess the effects of all classes of protein sequence variations beyond single amino acid substitutions, including in-frame indels and multiple amino acid substitutions.

### Using different protein databases to generate the supporting sequence set

The prediction accuracy of PROVEAN is determined by the supporting set of homologous sequences, which are in turn determined by the choice of protein database and the method of sequence collection. In determining the optimal prediction accuracy, we have compared the performance of different protein databases and sequence collection methods. We have compared the performance of PROVEAN using two different protein databases, the NCBI NR (non-redundant) protein database and the UniProtKB/Swiss-Prot protein database. The UniProtKB/Swiss-Prot database contains manually reviewed and curated high-quality sequences. Our results showed a reduced accuracy of 7% when using the UniProtKB/Swiss-Prot database instead of the NCBI NR protein database. One speculation for the reduced performance is that despite the high-quality protein sequences of the UniProtKB/Swiss-Prot database, the number of orthologous and distantly related sequences are not as sufficiently represented as in the NCBI NR protein database. To support this idea, we examined a set of 7,547 human protein variants (out of a total of 58,684) that were incorrectly predicted when using the UniProtKB/Swiss-Prot but correctly predicted using the NCBI NR database. When using the UniProtKB/Swiss-Prot database, the average number of supporting sequences was only 51 for the incorrectly predicted variant set, which was lower than the average number of 73 supporting sequences for the rest of the human protein variants. We speculate that a lack of sequence information as represented by the small supporting sequence set could be one of the factors leading to a reduced accuracy when using the UniProtKB/Swiss-Prot database to generate PROVEAN predictions.

We also compared the prediction accuracy of PROVEAN by directly supplying precomputed ortholog datasets obtained from the Ensembl Compara or the OMA databases [23], [24]. Our results showed a reduced accuracy of 3–5% when using the precomputed orthologs. Finally, BLASTP and PSI-BLAST (Position-Specific Iterated BLAST) were compared for the collection of homologous and related sequences from the NCBI NR protein database. No significant difference in accuracy was observed when using BLASTP or PSI-BLAST.

### Number of supporting sequences used and prediction accuracy

**Figure 9:**
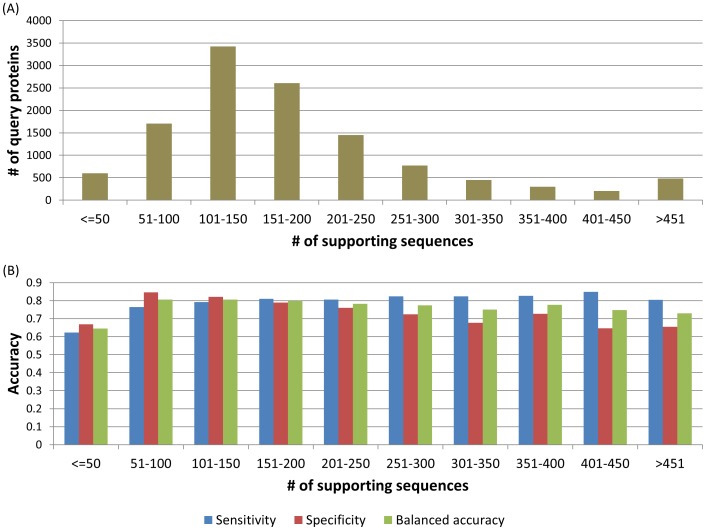
Number of supporting sequences used for the Uniprot human proteins carrying neutral or deleterious variants. (A) Distribution of the 11,990 human proteins based on the number of supporting sequences used for PROVEAN prediction. (B) Prediction accuracy achieved with respect to the number of supporting sequences. The observed accuracy is consistently above 73%, except in cases when the number of supporting sequences drops below 50.

In order to demonstrate how many supporting sequences are typically used for the prediction and whether prediction accuracy depends on the number of supporting sequences, we counted the number of supporting sequences used in generating PROVEAN predictions for the 11,990 human protein dataset carrying sequence variants. Our results showed that the majority uses 100–200 supporting sequences (average 229 sequences; median 155 sequences) (Figure 9A). The results also showed that the balanced accuracy was consistently above 73% regardless of the number of supporting sequences used except in cases when the number of supporting sequences drops below 50 (Figure 9B).

### Unbiased averaged delta alignment score

Different approaches can be used to combine delta alignment scores and compute a final PROVEAN score from a set of supporting sequences to generate PROVEAN prediction (e.g. simple average, weighted average). The approach we took was to first cluster the protein sequences by sequence similarity, then compute a within-cluster average delta score for each cluster, and finally compute a between-cluster average delta score among the clusters so that all clusters are weighted equally (see Methods). This approach of clustering and averaging prevents the final delta score from being biased by a potentially large set of closely related sequences that are overrepresented in a protein database. We have also tested different weighting schemes systematically based on different sequence similarity of the sequence clusters with respect to the protein query (e.g. use higher weights for clusters more similar to the query). However, we found that even with more complicated weighting schemes, the overall accuracy was not increased significantly.

### Genome-wide application

Exome capture and high-throughput sequencing has become a popular approach to discover and associate genetic variants with common genetic diseases [25], [26], [27], [28], [29], [30]. In such studies, genetic variants are filtered based on population allele frequency to remove commonly occurring polymorphisms in the human populations, and prediction tools for coding variants are used to provide assessments on the impact of the genetic variants on protein function [25], [27], [29]. For example, candidate disease genes can be prioritized by the total number of genetic variants that are predicted as deleterious on a given gene sequence. For these studies, PROVEAN can be used to identify deleterious single nucleotide variants and variants that cause protein sequence indels. In addition, the delta alignment score approach can potentially be expanded to prioritize candidate disease gene by combining the alignment scores across multiple point mutations along the protein sequence.

## Methods

### Dataset collection

The UniProt human protein variation dataset used in this study contains a single amino acid substitution dataset and a one to six amino acid in-frame indel dataset. The human single amino acid substitution dataset was obtained from the UniProt “Human Polymorphisms and Disease Mutations” dataset (Release 2011_09; http://www.uniprot.org/docs/humsavar), which contains 20,821 pre-annotated disease variants and 36,825 common polymorphisms. The human small in-frame indel dataset was built in-house from additional types of natural variations including deletions, insertions, and replacements (in-frame substitution of multiple amino acids) of length up to 6 amino acids from the UniProtKB/Swiss-Prot database (Dataset S1). Each variant was annotated as deleterious, neutral, or unknown based on keywords found in the biological feature descriptions in the UniProt record using an automated script. Keywords such as “inhibit,” “affect,” “abolish,” and “loss” are classified as deleterious and keywords such as “does not affect,” “no loss,” and “does not reduce” are classified as neutral. A total of 729, 171, and 138 human protein variations of deletions, insertions, and replacements were collected, respectively.

The UniProt non-human protein variation dataset was created in-house from non-human organisms including viruses, fungi, bacteria, plants, etc. available in UniProtKB/Swiss-Prot (Release 2011_09). The non-human dataset contains single amino acid substitution and one to six amino acid in-frame indel datasets. Each variant was annotated as deleterious, neutral, or unknown using the same approach as in the UniProt human protein variation dataset.

The HGMD small in-frame indel dataset was collected from the HGMD database (Professional ver2011.3). The 1000 Genomes small in-frame indel dataset (Dataset S2) was collected from the 1000 Genomes Project (August 2010 release, ftp://ftp.1000genomes.ebi.ac.uk/vol1/ftp/release/20100804). Indels classified as disease-causing mutations in the HGMD were first collected. This dataset was further filtered to collect small in-frame indels leading to one to six amino acid insertions or deletions using Mutalyzer (https://mutalyzer.nl/) [31]. Indels were also collected from the 1000 Genomes Project using a similar approach. A total of 10 indels were represented in both the 1000 Genomes and the HGMD datasets and were considered as deleterious.

The *E. coli* LacI amino acid mutation screening data was originally generated by Markiewicz et al. [32]. In their study, a total of 4,041 single amino acid substitutions of the *E. coli* lactose operon repressor protein were assayed for the effect on LacI function, and the resulting phenotypes were classified. For our binary classification test, a compiled version of the mutation for LacI function dataset was obtained from http://blocks.fhcrc.org/sift/test_sets/ (Table S3) [5].

For the human tumor suppressor protein TP53, a set of 2,314 single point mutants and corresponding biological activity levels were obtained from the IARC TP53 database [33]. The TP53 mutants had been functionally classified into four classes—non-functional, partially-functional, functional (wild-type), and supertrans (higher than wild-type activity)—based on the median of 8 promoter-specific activity levels. The functional assays were performed in yeast by Kato et al. [18] and protein activity was measured as percentage of wild-type activity. In the current comparison, the “non-functional” mutations were classified as deleterious variants and the other three functional mutation classes were considered neutral variants (Table S4).

The ABCA1 protein mutants were created by Brunham et al. [19] for a total of 17 single amino acid substitutions. Cholesterol efflux was measured in all mutants and wild-type to assess ABCA1 function (Table S2).

### PROVEAN prediction

**Figure 10:**
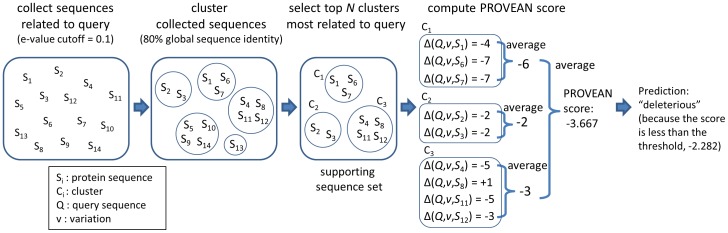
Computing the PROVEAN score. For simplicity, only the top three clusters were included in building the supporting sequence set in this example.

PROVEAN consists of two main steps (a detailed flowchart in Figure S3). In the first step, PROVEAN collects a set of homologous and distantly related sequences from the NCBI NR protein database (released August 2011) using BLASTP (ver.2.2.25) with an E-value threshold of 0.1. The sequences are clustered based on a sequence identity of 80% to remove redundancy using the CD-HIT program (ver.4.5.5) [34]. Starting from the sequence cluster most similar to the query sequence, the clusters are added to the supporting sequence set one by one until there is a sufficient number of clusters in the supporting set. We currently used a cutoff of 45 clusters, that is, all sequences from up to 45 clusters are used as the supporting sequence set. In the second step, for each sequence in the supporting sequence set, a delta score is computed using the BLOSUM62 substitution matrix and gap penalties of 10 for opening and 1 for extension. Within each cluster, an average delta score is computed. The averaged delta scores are again averaged among all clusters so that each cluster is weighted equally. This unbiased averaged delta score is the final PROVEAN score. The equation for computing the unbiased averaged delta score is shown in the equation below:
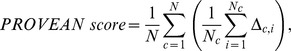
where 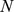 is the number of clusters in the supporting set, 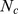 is the number of supporting sequences in the *c*-th cluster, and 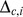 is the delta score with respect to the *i*-th supporting sequence in the *c*-th cluster. An example of computing a PROVEAN score is shown in Figure 10. If the PROVEAN score is smaller than or equal to a given threshold, the variation is predicted as deleterious.

### Parameter selection for delta score computation

Parameters for computing delta scores include the choice of log-odds scores from substitution matrix, gap penalties, percent identity threshold for sequence clustering, and the number of top clusters (most similar to query) to include supporting homologous sequences for delta alignment score computation. These parameters were optimized for the best performance of PROVEAN. Here we describe how the parameters were selected.

For the substitution matrix, the BLOSUM62 matrix was chosen since it is one of the most commonly used matrices. Our preliminary results showed that the performance of PROVEAN was similar when using other substitution matrices (BLOSUM80, PAM matrices, and RBLOSUM64 [35]). The remaining three parameters (gap penalties, percent identity, and number of clusters) were tested systematically for a restricted number of combinations to avoid over-fitting. The best balanced separation of the deleterious and neutral classes in the UniProt human protein variation dataset (Table 1) was measured in terms of balanced accuracy for each of 756 different combinations of parameter values (6 different values for gap penalties, 7 for percent identity for clustering, and 18 for number of clusters). The parameter combination that gave the highest accuracy of 79.05% was chosen as the default parameter values. However, we observed that for a wide range of combinations the accuracy was highly similar (Figure S4, Table S5). The optimal parameters for gap opening/extension penalties, percent identity, and number of clusters are 10/1, 80%, and 45, respectively.

### Comparison with other prediction tools

Several prediction tools provide classification of coding variants into more than two classes. Mutation Assessor classifies variants into four classes (high impact, medium impact, low impact, and neutral). In the current comparison, we used two thresholds, 0.8 (between neutral class and the rest) and 1.9 (between low impact and medium impact classes) to compute accuracy. PolyPhen-2 generates three outcomes of predictions (probably damaging, possibly damaging, and benign). In the current comparison, the threshold of 0.432 (between benign class and the other two damaging classes) was used to compute accuracy.

To obtain SIFT predictions, we installed the SIFT program (ver.4.0.3, http://sift.jcvi.org/) locally and ran it using the NCBI NR protein database (released August 2011). For other prediction tools, we generated prediction output using the corresponding public web servers and published score threshold as suggested by the authors. The PolyPhen-2 web server (http://genetics.bwh.harvard.edu/pph2/) supports version 2.1.0 and uses protein sequences from UniProtKB/UniRef100 Release 2011_0, and protein structures from PDB/DSSP Snapshot 06-Apr-2011. The HumVar model was used for generating prediction results for the LacI and TP53 datasets. Since the HumVar model was originally trained with UniProt human variations and most of which overlapped with our datasets, the HumDiv model was used to generate PolyPhen-2 predictions for our UniProt human and non-human protein variation datasets. The Mutation Assessor web server (version 1.0, http://mutationassessor.org/v1/) uses Pfam 25 (March 2011), PDB (August 2011), UniProtKB/Swiss-Prot and UniProtKB/TrEMBL (2011_05). The Condel scores for human protein variations were obtained from the Condel web server, which integrates the outputs of SIFT, PolyPhen-2, and Mutation Assessor (version 1.4, http://bg.upf.edu/condel/) and provides a consensus prediction. The MAPP scores for LacI and TP53 were obtained from the supplementary data of the original publication (http://mendel.stanford.edu/supplementarydata/stone_MAPP_2005/).

## Supporting Information

Figure S1 **PROVEAN scores were generated for all possible single amino acid substitutions, deletions, and insertions at each position in the human protein TP53.** The scores are represented as a color intensity scale from −18 to 5 (bottom right). For substitutions and insertions, each row represents one of 20 amino acids in the variant. The amino acid residues are grouped by polarity and charge. From the top, polar acidic (D,E), polar basic (H,R,K), polar uncharged (Q,N,Y,C,T,S,G), and non-polar hydrophobic (A,V,L,I,F,W,M,P). In general, low PROVEAN scores are found in conserved regions or domains, and high scores are found in non-conserved regions.
(TIF)

Figure S2 **Correlation of the PROVEAN score and median transactivation activity level of human TP53.** Each dot represents a point mutation of the TP53 protein. The dataset contains 2,314 single amino acid mutants and activities on eight p53 response-elements measured in a yeast assay (Pearson's correlation coefficient of 0.556). TP53 mutation and activity originally produced in [18].
(TIF)

Figure S3 **A flowchart to describe the PROVEAN procedure.**
(TIF)

Figure S4 **Balanced accuracy for different parameter values for clustering with fixed gap penalties of 10 for opening and 1 for extension.** The highest accuracy, 79.05%, was achieved at the combination of 45 clusters and 80% identity denoted by an arrow. The accuracy is higher than 78% for a wide range of parameter combinations.
(TIF)

Table S1 Binary classification performance of Condel for single amino acid substitutions in human proteins.
(DOCX)

Table S2 PROVEAN score and cholesterol efflux for ABCA1 variations.
(DOCX)

Table S3 LacI mutation dataset used for assessing PROVEAN performance.
(DOCX)

Table S4 TP53 mutation dataset used for assessing PROVEAN performance.
(DOCX)

Table S5 Assessment of different parameter combinations for PROVEAN.
(DOCX)

Dataset S1 **Human indel variants collected from the UniProt/Swiss-Prot database.**
(XLSX)

Dataset S2 **Human indel variants collected from the 1000 Genomes Project.**
(XLSX)
