# Supplementary figures and images for: Predicting the Functional Effect of Amino Acid Substitutions and Indels
Source: PLoS One. 2012 Oct 8;7(10):e46688. doi: 10.1371/journal.pone.0046688 (PMC3466303; doi:10.1371/journal.pone.0046688)

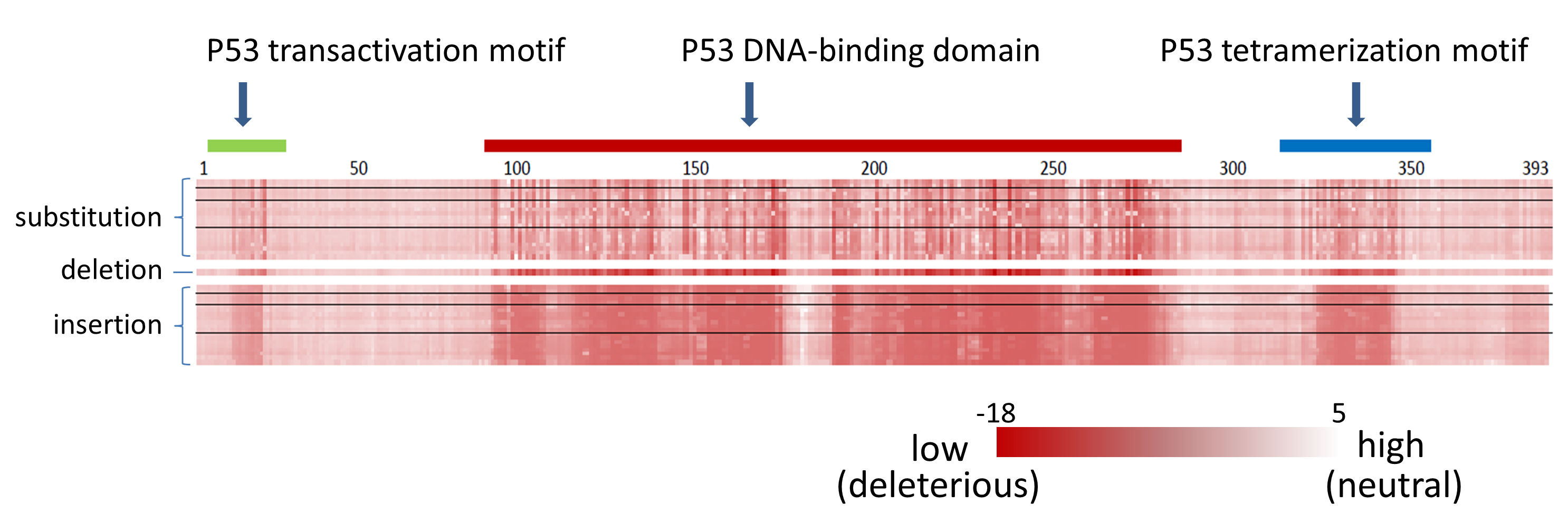

Supplement: Figure S1 — PROVEAN scores were generated for all possible single amino acid substitutions, deletions, and insertions at each position in the human protein TP53. The scores are represented as a color intensity scale from −18 to 5 (bottom right). For substitutions and insertions, each row represents one of 20 amino acids in the variant. The amino acid residues are grouped by polarity and charge. From the top, polar acidic (D,E), polar basic (H,R,K), polar uncharged (Q,N,Y,C,T,S,G), and non-polar hydrophobic (A,V,L,I,F,W,M,P). In general, low PROVEAN scores are found in conserved regions or domains, and high scores are found in non-conserved regions. (TIF) [file pone.0046688.s001.tif]

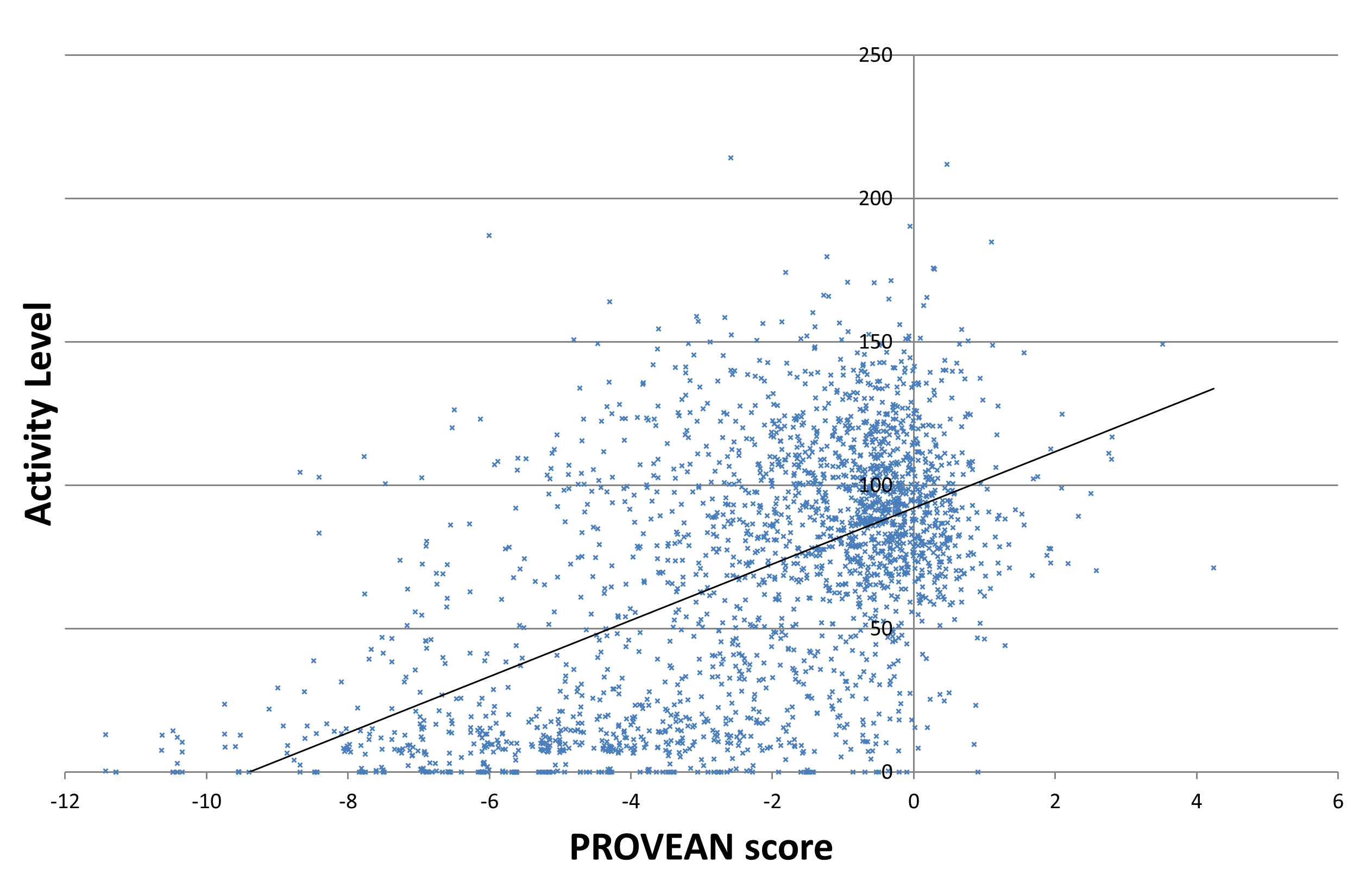

Supplement: Figure S2 — Correlation of the PROVEAN score and median transactivation activity level of human TP53. Each dot represents a point mutation of the TP53 protein. The dataset contains 2,314 single amino acid mutants and activities on eight p53 response-elements measured in a yeast assay (Pearson's correlation coefficient of 0.556). TP53 mutation and activity originally produced in [18]. (TIF) [file pone.0046688.s002.tif]

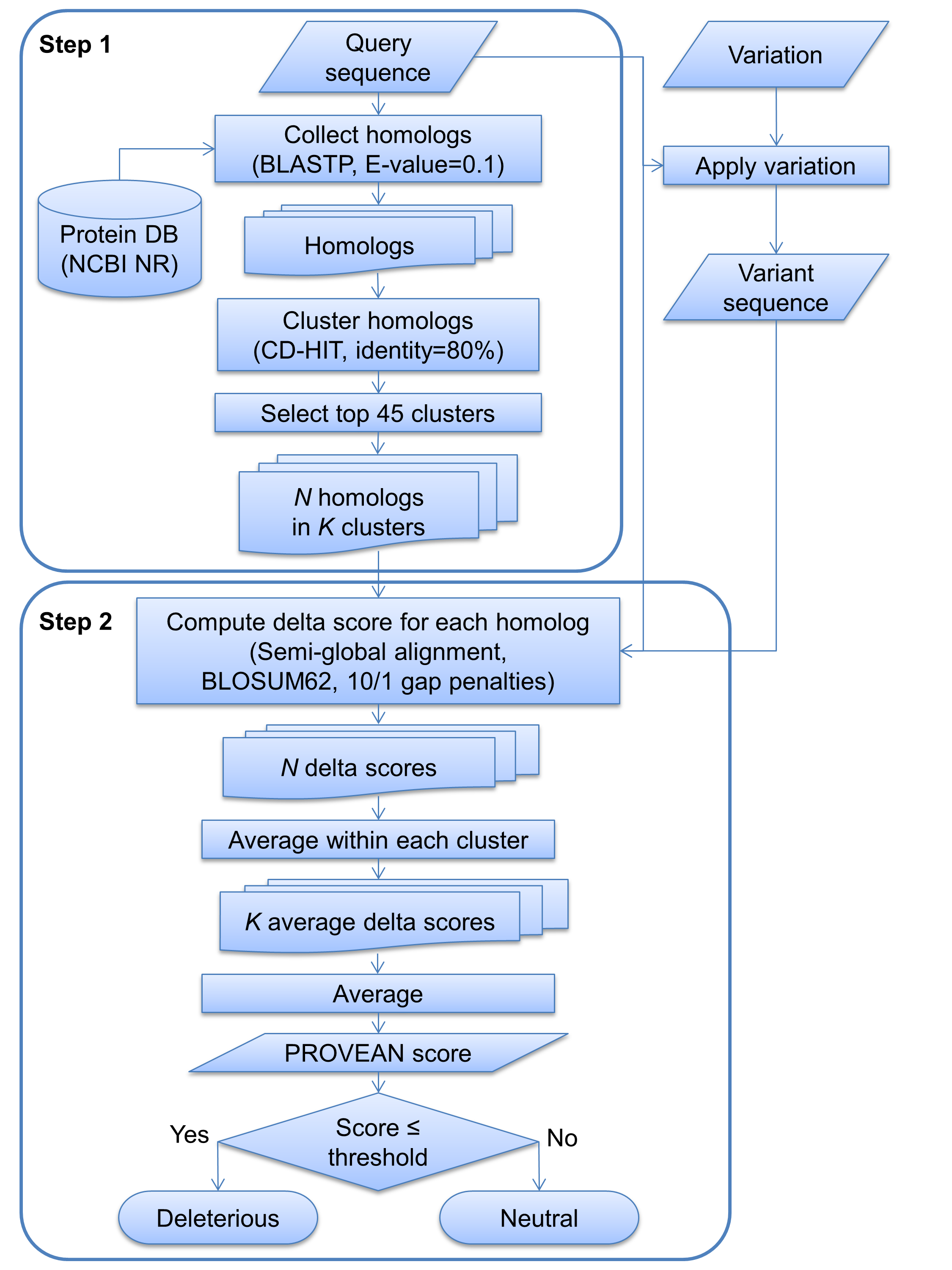

Supplement: Figure S3 — A flowchart to describe the PROVEAN procedure. (TIF) [file pone.0046688.s003.tif]

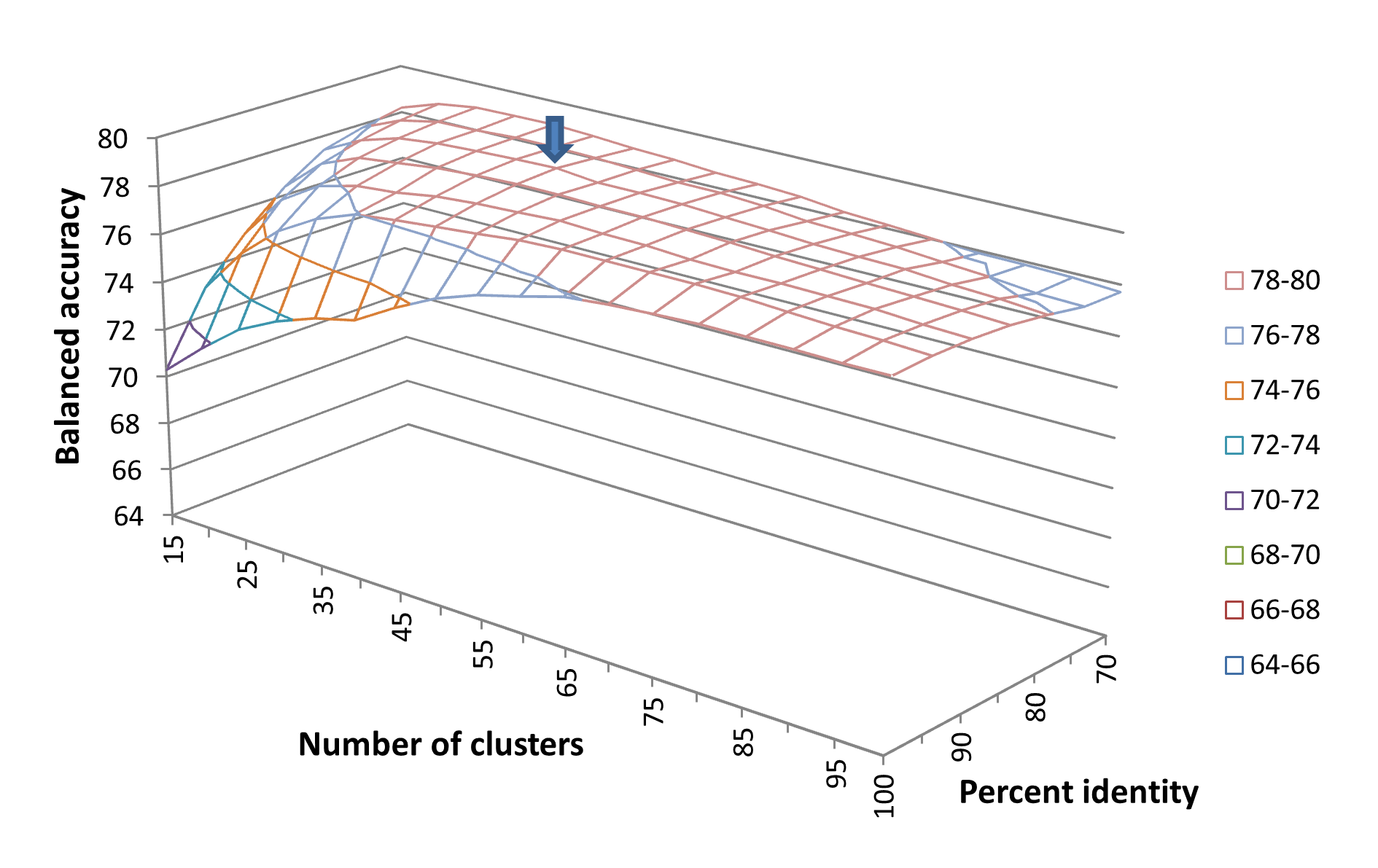

Supplement: Figure S4 — Balanced accuracy for different parameter values for clustering with fixed gap penalties of 10 for opening and 1 for extension. The highest accuracy, 79.05%, was achieved at the combination of 45 clusters and 80% identity denoted by an arrow. The accuracy is higher than 78% for a wide range of parameter combinations. (TIF) [file pone.0046688.s004.tif]
