# Supplementary material for: Predicting the Functional Effect of Amino Acid Substitutions and Indels
Source: PLoS One. 2012 Oct 8;7(10):e46688. doi: 10.1371/journal.pone.0046688 (PMC3466303; doi:10.1371/journal.pone.0046688)
Supplement: Table S1 — Binary classification performance of Condel for single amino acid substitutions in human proteins. (DOCX) [file pone.0046688.s005.docx]

**Table S1**. Binary classification performance of Condel for single amino acid substitutions in human proteins.

| Threshold | Balanced accuracy | Sensitivity | Specificity | No prediction | Error |
| --- | --- | --- | --- | --- | --- |
| 0.469 | 70.04 | 93.84 | 46.23 | 1499 (2.60%) | 5695 (9.88%) |
| 0.790 | 75.66 | 75.69 | 75.63 |  |  |

Results from two thresholds were compared: (1) the default threshold of 0.469 as used in the Condel web server, and (2) a threshold of 0.790 to maximize the balanced accuracy. “No prediction” column shows the number of variations for which the web server outputs “NA.” The “Error” column shows the number of variations for which the web server generates no output.
