# Supplementary material for: Predicting the Functional Effect of Amino Acid Substitutions and Indels
Source: PLoS One. 2012 Oct 8;7(10):e46688. doi: 10.1371/journal.pone.0046688 (PMC3466303; doi:10.1371/journal.pone.0046688)
Supplement: Table S2 — PROVEAN score and cholesterol efflux for ABCA1 variations. (DOCX) [file pone.0046688.s006.docx]

Table S2. PROVEAN score and cholesterol efflux for ABCA1 variations.

| Variant | Cholesterol Efflux (%) | | | PROVEAN Score | PROVEAN Prediction | Disease association from UniProt |
| --- | --- | --- | --- | --- | --- | --- |
| C1477R | 20.5 | ± | 10 | -9.137 | Deleterious | disease |
| R2081W | 21.1 | ± | 21 | -6.381 | Deleterious | disease |
| W590S | 47.1 | ± | 13 | -6.276 | Deleterious | disease |
| A1046D | 16.8 | ± | 7 | -5.123 | Deleterious | disease |
| S1506L | 17.8 | ± | 15 | -4.701 | Deleterious | disease |
| M1091T | 6.9 | ± | 20 | -4.590 | Deleterious | disease |
| R587W | 31.7 | ± | 33 | -4.350 | Deleterious | disease |
| T929I | 69.9 | ± | 11 | -4.301 | Deleterious | disease |
| N935S | 29.3 | ± | 13 | -4.194 | Deleterious | disease |
| P2150L | 88.4 | ± | 21 | -3.984 | Deleterious | disease |
| S1731C | 12.3 | ± | 10 | -3.630 | Deleterious | polymorphism |
| Q597R | 17.7 | ± | 14 | -3.287 | Deleterious | disease |
| N1800H | 31.3 | ± | 16 | -2.863 | Deleterious | disease |
| R219K | 103.7 | ± | 21.05 | -0.084 | Neutral | polymorphism |
| D1289N | 137.7 | ± | 86 | 0.115 | Neutral | disease |
| I883M | 69.1 | ± | 16 | 0.257 | Neutral | polymorphism |
| V771M | 145.4 | ± | 33 | 0.525 | Neutral | polymorphism |
| Wild-type | 100 |  |  | 0 |  |  |

A total of 15 mutants (88%) were correctly predicted by PROVEAN with reference to UniProtKB/Swiss-Prot disease versus common polymorphism classification of the amino acid variants.
