# Supplementary material for: Predicting the Functional Effect of Amino Acid Substitutions and Indels
Source: PLoS One. 2012 Oct 8;7(10):e46688. doi: 10.1371/journal.pone.0046688 (PMC3466303; doi:10.1371/journal.pone.0046688)
Supplement: Table S3 — LacI mutation dataset used for assessing PROVEAN performance. (DOCX) [file pone.0046688.s007.docx]

**Table S3.** LacI mutation dataset used for assessing PROVEAN performance.

| Class | # of substitutions | Meaning | Binary class used for PROVEAN assessment |
| --- | --- | --- | --- |
| - | 1166 | affecting | deleterious |
| -+ | 355 | affecting, not as severe as '-' | deleterious |
| +- | 253 | affecting, not as severe as '-+' | deleterious |
| + | 2267 | not altering phenotype | neutral |
| Total | 4041 |  |  |

Note: LacI mutation dataset originally produced by Markiewicz et al. [1].

**Reference**

1. Markiewicz P, Kleina LG, Cruz C, Ehret S, Miller JH (1994) Genetic studies of the lac repressor. XIV. Analysis of 4000 altered Escherichia coli lac repressors reveals essential and non-essential residues, as well as "spacers" which do not require a specific sequence. J Mol Biol 240: 421-433.
