# Supplementary material for: Predicting the Functional Effect of Amino Acid Substitutions and Indels
Source: PLoS One. 2012 Oct 8;7(10):e46688. doi: 10.1371/journal.pone.0046688 (PMC3466303; doi:10.1371/journal.pone.0046688)
Supplement: Table S4 — TP53 mutation dataset used for assessing PROVEAN performance. (DOCX) [file pone.0046688.s008.docx]

Table S4. TP53 mutation dataset used for assessing PROVEAN performance.

| Class | # of substitutions | Median activity level | Binary class used for PROVEAN assessment |
| --- | --- | --- | --- |
| Supertrans (Increased activity) | 85 | > 140 | neutral |
| Functional | 1154 | > 75 and <= 140 | neutral |
| Partially functional | 582 | >20 and <=75 | neutral |
| Non-functional | 493 | <= 20 | deleterious |
| Total | 2314 |  |  |

Note: TP53 mutation dataset originally produced by Kato et al. [1].

**Reference**

1. Kato S, Han SY, Liu W, Otsuka K, Shibata H, et al. (2003) Understanding the function-structure and function-mutation relationships of p53 tumor suppressor protein by high-resolution missense mutation analysis. Proc Natl Acad Sci U S A 100: 8424-8429.
