# Supplementary material for: Predicting the Functional Effect of Amino Acid Substitutions and Indels
Source: PLoS One. 2012 Oct 8;7(10):e46688. doi: 10.1371/journal.pone.0046688 (PMC3466303; doi:10.1371/journal.pone.0046688)
Supplement: Table S5 — Assessment of different parameter combinations for PROVEAN. (DOCX) [file pone.0046688.s009.docx]

Table S5. Assessment of different parameter combinations for PROVEAN.

| Gap penalties (open, extension) | Balanced accuracy |
| --- | --- |
| 10,1 | 79.05 |
| 11,1 | 79.02 |
| 12,1 | 79.01 |
| 7,2 | 78.98 |
| 8,2 | 78.94 |
| 9,2 | 78.94 |

Balanced accuracy is shown when testing different gap penalties with other parameters fixed (45 clusters with 80% clustering threshold). No significant change in balanced accuracy was observed with the parameter combinations tested.
